# Test–retest reliability of self-reported diabetes diagnosis in the Norwegian Women and Cancer Study: A population-based longitudinal study (n =33,919)

**DOI:** 10.1177/2050312115622857

**Published:** 2016-01-08

**Authors:** Mashhood Ahmed Sheikh, Eiliv Lund, Tonje Braaten

**Affiliations:** Department of Community Medicine, University of Tromsø, Tromsø, Norway

**Keywords:** Type 2 diabetes, type 1 diabetes, metabolic syndrome, kappa, test–retest reliability, reproducibility, questionnaires, Norway, Norwegian Women and Cancer, Kvinner og kreft

## Abstract

**Objective:**

Self-reported information from questionnaires is frequently used in epidemiological studies, but few of these studies provide information on the reproducibility of individual items contained in the questionnaire. We studied the test–retest reliability of self-reported diabetes among 33,919 participants in Norwegian Women and Cancer Study.

**Methods:**

The test–retest reliability of self-reported type 1 and type 2 diabetes diagnoses was evaluated between three self-administered questionnaires (completed in 1991, 1998, and 2005 by Norwegian Women and Cancer participants) by kappa agreement. The time interval between the test–retest studies was ~7 and ~14 years. Sensitivity of the kappa agreement for type 1 and type 2 diabetes diagnoses was assessed. Subgroup analysis was performed to assess whether test–retest reliability varies with age, body mass index, physical activity, education, and smoking status.

**Results:**

The kappa agreement for both types of self-reported diabetes diagnoses combined was good (⩾0.65) for all three test–retest studies (1991–1998, 1991–2005, and 1998–2005). The kappa agreement for type 1 diabetes was good (⩾0.73) in the 1991–2005 and the 1998–2005 test–retest studies, and very good (0.83) in the 1991–1998 test–retest study. The kappa agreement for type 2 diabetes was moderate (0.57) in the 1991–2005 test–retest study and good (⩾0.66) in the 1991–1998 and 1998–2005 test–retest studies. The overall kappa agreement in the 1991–1998 test–retest study was stronger than in the 1991–2005 test–retest study and the 1998–2005 test–retest study. There was no clear pattern of inconsistency in the kappa agreements within different strata of age, BMI, physical activity, and smoking. The kappa agreement was strongest among the respondents with 17 or more years of education, while generally it was weaker among the least educated group.

**Conclusion:**

The test–retest reliability of the diabetes was acceptable and there was no clear pattern of inconsistency in the kappa agreement stratified by age, body mass index, physical activity, and smoking. The study suggests that self-reported diabetes diagnosis from middle-aged women enrolled in the Norwegian Women and Cancer Study is reliable.

## Introduction

Epidemiological studies often rely on self-reported information, as this renders the costs of data collection lower than that of clinical studies.^1^ However, the validity and reliability of the instruments used for data collection are often not reported.^2^

Commonly, the Cohen’s kappa coefficient is used to determine inter-rater agreement for disease (or other categorical outcomes) by comparing self-reported information against a gold standard (diagnostic test, medical records, physiological measures, etc.). Previous validation studies of self-reported diabetes diagnosis have indicated that diabetes is reported more accurately than other illnesses or diseases.^3–10^

The Cohen’s kappa coefficient can also be used to analyze the test–retest reliability of an instrument. Many studies from Norway have used self-reported information from questionnaires as the principle tool, but few^11–43^ of them have provided information on the reproducibility of the individual items and instruments therein. It is important to establish that respondents with different socio-demographic background, and age groups have understood the questions in a similar manner. Test–retest reliability is assessed by measuring the responses of the same study sample to an identical question at two or more points in time.^44^ These responses are then compared to establish the reliability of the instrument. The chi-square (χ^2^) test for independence is not appropriate for assessing test–retest reliability since it does not take into account that the data are paired (i.e. different measurements for the same individual).

Previous studies using self-reported data from interviews have studied the test–retest reliability of self-reported diabetes diagnosis, with inconsistent kappa agreements.^45–50^ Since type 2 diabetes typically affects people aged 40 years and over,^51,52^ it is possible to differentiate between the test–retest reliability of self-reported type 1 and type 2 diabetes diagnoses using information on age at diagnosis. No previous study was found that assessed the test–retest reliability for either type 1 or type 2 diabetes separately.

The Norwegian Women and Cancer (NOWAC) Study^53^ is a prospective cohort study in which women reported diabetes diagnosis and age at diagnosis in three separate questionnaires. If a woman accurately reported her diabetes diagnosis in one study, she is expected to report the same in a subsequent study. This assumption underlies our test–retest reliability analysis. The aim of this study was to assess the test–retest reliability of self-reported diabetes diagnosis, as well as that of type 1 and type 2 diabetes diagnoses separately. Furthermore, the large sample size permits subgroup analyses and sensitivity analysis. We examined whether test–retest reliability varies with age, body mass index (BMI), physical activity, education, and smoking status.

## Methods

### Study cohort and sampling

The NOWAC Study is a prospective nationwide study which started in 1991,^54,55^ and contains data from 170,000 women. Participants were randomly selected from the National Population Register of Norway. The external validity of the study^56^ and validity of some measures^57–59^ have been published elsewhere. NOWAC Study participants are assumed to be representative of the female Norwegian population in the corresponding age groups.^56^ The detailed characteristics of the participants are described elsewhere,^56^ and the updated information on the NOWAC Study is accessible on its website.^54^

Of the 170,000 women enrolled in the NOWAC Study, 33,919 women completed all of three questionnaires sent in 1991, 1998, and 2005. The general characteristics of the study sample and the association between BMI and type 2 diabetes in this sample are described elsewhere.^52^

### Questionnaire and classification

#### Diabetes

Information on diabetes diagnosis was collected by means of the same question in all three questionnaires (1991, 1998, and 2005): “Have you had any of the following diseases?” The list of options included diabetes. Age at diagnosis was measured with the subsequent question, “If yes, at what age was it first discovered?” For the purposes of this study, only participants who reported having diabetes and provided their age at diagnosis were defined as diabetes cases. If participants reported they gave birth to a child either the same year they were diagnosed with diabetes, or in the year preceding child birth, it was assumed that they had gestational diabetes, and they were excluded from the analysis. Final numbers of diabetes cases included in analyses are given in Tables 2–4. Participants with missing values on diabetes diagnosis and age at diagnosis were excluded.

Using the responses to the questions on diabetes and age at diagnosis, different variables for diabetes diagnosis, and separate variables for type 1 and type 2 diabetes, were created. Since type 2 diabetes typically affects people aged 40 years or over,^51,52^ we classified only those aged 40 years or over as having type 2 diabetes. Women who were diagnosed with diabetes at or before age 39 years were categorized as having type 1 diabetes (excluding those with gestational diabetes). Participants with type 1 and type 2 diabetes were classified separately by the above-mentioned criteria for the 1991 test study, the 1998 test study, the 1998 retest study for comparison against 1991 test study, the 2005 retest study for comparison against the 1991 test study, and the 2005 retest study for comparison with the 1998 test study.

Diabetes cases in the 1991 and 1998 test studies were defined as those who reported having diabetes, and their age at diagnosis in the corresponding questionnaires. One respondent to the 1998 questionnaire fulfilled the criteria for both gestational diabetes and type 2 diabetes and was finally classified as having gestational diabetes only.

#### Diabetes in the 1998 retest study (for comparison against the 1991 test study)

Diabetes cases in the 1998 retest study, for comparison against the 1991 test study were defined as those with diabetes from the 1998 test study, provided they reported a date of diagnosis prior to 1992. The same criteria were applied to women with type 1 or type 2 diabetes. One women in the 1998 retest study fulfilled the criteria both for gestational and type 2 diabetes and was finally classified as having gestational diabetes only.

#### Diabetes in the 2005 retest study (for comparison against the 1991 test study)

Diabetes cases from the 2005 retest study, for comparison against 1991 test study, were defined as participants who reported a diabetes diagnosis in the 2005 questionnaire, provided they reported a date of diagnosis prior to 1992. The same criteria were applied to women with type 1 or type 2 diabetes.

#### Diabetes in the 2005 retest study (for comparison against 1998 test study)

Diabetes cases from the 2005 retest study, for comparison against the 1998 test study, were defined as participants with self-reported diabetes in the 2005 questionnaire, provided that they reported a date of diagnosis prior to 1999. The same criteria were applied to women with type 1 or type 2 diabetes.

#### Covariates

Self-reported information on height and weight from 1998 study was used to calculate BMI (kg/m^2^). BMI was categorized into three groups: normal weight (BMI: <25 kg/m^2^), overweight (BMI: 25–29.9 kg/m^2^), and obese (BMI: ⩾30 kg/m^2^). Smoking status was derived from the replies to two questions in the 1998 questionnaire: “Have you ever smoked?” (yes, no) and “Do you smoke on a daily basis at the moment?” (yes, no). Women who answered “no” to the former were categorized as “never smokers.” Those who answered “yes” to the former, and “no” to the latter, were categorized as “former smokers,” and those who answered “yes” to both questions were categorized as “current smokers.” A 10-category scale measured the level of self-reported physical activity in the 1998 questionnaire, the validity of which has been reported previously.^21^ Responses to questions about physical activity were used to assign a category of physical activity: low [1–3], medium [4–7], and high [8–10]. Education (duration in years) was categorized into four groups: primary/intermediate (0–9), secondary (10–12), university (13–16), and postgraduate and above (17+). Age (years) was categorized in four groups with 5-year interval.

### Statistical analysis

Statistical analysis was performed with SAS version 9.2 and Stata version 13.1. Means (standard deviation (SD)) were estimated for all continuous variables, and the percentage of participants in each category was calculated for all categorical variables. General characteristics of the data are presented as frequencies, percentages, and means with SDs, respectively (Table 1). Variables for all diabetes diagnoses, as well as for type 1 and type 2 diabetes separately, were constructed, and the kappa agreement for the two types of diabetes was calculated for the 1991–1998 test–retest study, the 1991–2005 test–retest study, and 1998–2005 test–retest study, respectively. The kappa coefficients summarize the total agreement beyond that expected by chance. 95% confidence intervals (CIs) for kappa statistic were estimated with analytical method^60^ in Stata.^61^ Established benchmarks^62,63^ for rating the strength of kappa agreements as poor (<0.20), fair (>0.20 to ⩽0.40), moderate (>0.40 to ⩽0.60), good (>0.60 to ⩽0.80), and very good (>0.80 to ⩽1.00) were used.

**Table 1.** General characteristics of the study sample (n = 33,919).

	Cohort n = 33,919	
	N (%)	Mean (SD)
Age (years)		47.7 (4.3)
40–44	9926 (29.3)	
45–49	11,382 (33.6)	
50–54	10,849 (32.0)	
55–59	1762 (5.2)	
BMI^a^		24.4 (3.8)
Normal weight (<25 kg/m^2^)	21,553 (64.6)	
Overweight (25–29.9 kg/m^2^)	9106 (27.3)	
Obese (⩾30 kg/m^2^)	2709 (8.1)	
Education level (duration in years)^a^		12.5 (3.2)
Primary/intermediate (0–9)	6736 (20.1)	
Secondary (10–12)	12,102 (36.1)	
University (13–16)	10,226 (30.5)	
Postgraduate and above (17+)	4460 (13.3)	
Physical activity level^a^		5.6 (1.7)
Low	3686 (11.5)	
Medium	24,229 (75.5)	
High	4186 (13.0)	
Smoking status		
Never smoker	13,763 (40.6)	
Former smoker	10,582 (31.2)	
Current smoker	9574 (28.2)	

SD: standard deviation; BMI: body mass index.

a Cohort size was 33,919, but because of missing values, the numbers for some variables do not add up to 33,919.

Consistency (%) was calculated as

$$\mathrm{Consistency}(\%)=\frac{\mathrm{Number of diabetes diagnosis in both test and retest study}}{\mathrm{Number of diabetes diagnosis in test study}}\times 100$$

### Sensitivity analysis

Since self-reported age at diagnosis was used as the only discriminative criterion for distinguishing between type 1 and type 2 diabetes, sensitivity analysis was performed by restricting age at diagnosis <35 years for type 1 diabetes and age at diagnosis >44 years for type 2 diabetes (Table 5). Those reporting age at diagnosis 35-44 were excluded for the purpose of assessing sensitivity of the kappa agreements (Table 5).

### Subgroup analysis

Subgroup analysis was performed to assess the consistency of the kappa agreement across stratas of the covariates (Tables 6–10).

### Ethical approval

The NOWAC Study was approved by the Regional Committee for Medical and Health Research Ethics. All participating women gave written informed consent.

## Results

Table 1 presents the general characteristics of the study sample. Among the 33,919 women participating in 1991, 1998, and 2005 study, the age distribution was between 40 and 59 (mean: 47.7 ± 4.3) in 1998. Majority (64.6%) of the respondents had normal weight (BMI: <25 kg/m^2^). Almost 40.3% of the respondents had some university education or more. Most (75.5%) of the respondents were classified as having medium level of physical activity. In this study sample, 28.2% were classified as being current smoker, while 31.2% were classified as being former smokers.

Table 2 presents the self-reported diabetes diagnosis in 1991 study, and 1998 study by self-reported age at diagnosis in respective studies. Majority (56%) of the self-reported diabetics reported age at diagnosis as 30 years or over in 1991 study, while over 64.7% reported age at diagnosis as 40 years or over in the 1998 study. This may partly be due to the aging cohort itself.

**Table 2.** Self-reported diabetes diagnosis in 1991 and 1998 test studies by self-reported age at diagnosis.

	Age groups	Diabetic in 1991^a^	Diabetic in 1998^b^
		n (%)	n (%)
Age at diagnosis	0–4	5 (3.4)^c^	4 (1.3)^d^
	5–9	10 (6.8)^c^	6 (1.9)^d^
	10–14	18 (12.2)^c^	17 (5.4)^d^
	15–19	10 (6.8)^c^	11 (3.5)^d^
	20–24	7 (4.7)^c^	12 (3.8)^d^
	25–29	15 (10.1)^c^	12 (3.8)^d^
	30–34	23 (15.5)^c^	19 (6.0)^d^
	35–39	23 (15.5)^c^	30 (9.5)^d^
	40–44	25 (16.9)^c^	75 (23.8)^d^
	45–49	12 (8.1)^c^	70 (22.2)^d^
	50–54	–	59 (18.7)^d^
	Total	148 (100.0)	315 (100.0)

a Diabetes cases in the 1991 test study were defined as those who reported having diabetes, and their age at diagnosis in the 1991 study.

b Diabetes cases in the 1998 test study were defined as those who reported having diabetes, and their age at diagnosis in the 1998 study. One respondent to the 1998 questionnaire fulfilled the criteria for both gestational diabetes and type 2 diabetes and was excluded.

c N and % of respondents reporting age at diagnosis in 1991 study.

d N and % of respondents reporting age at diagnosis in 1998 study.

Tables 3 and 4 present the kappa statistics for the test–retest studies. The agreement for all self-reported diabetes diagnoses in the 1991–1998 test–retest study was 0.75 (95% CI: 0.70–0.81), while it was 0.70 (95% CI: 0.66–0.74) in the 1998–2005 test–retest study. The kappa agreement for all self-reported diabetes diagnoses in the 1991–2005 test–retest study was 0.65 (95% CI: 0.58–0.71) (Table 3).

**Table 3.** Kappa agreements for self-reported diabetes diagnoses (excluding gestational diabetes).

	Test study	Retest study	Consistency (%)^a^	Kappa (95% CI)
Diabetes	Cases in 1991 (n)	Cases in 1998 (n)		
	148	151	113/148 (76.4)	0.75 (0.70–0.81)
	Cases in 1991 (n)	Cases in 2005 (n)		
	148	130	90/148 (60.8)	0.65 (0.58–0.71)
	Cases in 1998 (n)	Cases in 2005 (n)		
	315	282	209/315 (66.3)	0.70 (0.66–0.74)

CI: confidence interval.a

a $\mathrm{Consistency}(\%)=\frac{\mathrm{Number of diabetes diagnosis in both test and retest study}}{\mathrm{Number of diabetes diagnosis in test study}}\times 100.$

**Table 4.** Kappa agreements for self-reported type 1 and type 2 diabetes diagnoses.

Diabetes type	Test study	Retest study	Consistency (%)^a^	Kappa (95% CI)
	Cases in 1991 (n)	Cases in 1998 (n)		
Type 1 diabetes^b^	111	103	83/111 (74.7)	0.83 (0.76–0.89)
Type 2 diabetes^c^	37	48	29/37 (78.4)	0.67 (0.55–0.79)
	Cases in 1991 (n)	Cases in 2005 (n)		
Type 1 diabetes^b^	111	88	64/111 (57.6)	0.76 (0.68–0.84)
Type 2 diabetes^c^	37	42	21/37 (56.6)	0.57 (0.43–0.71)
	Cases in 1998 (n)	Cases in 2005 (n)		
Type 1 diabetes^b^	111	97	70/111 (63.1)	0.73 (0.66–0.81)
Type 2 diabetes^c^	204	185	125/204 (61.3)	0.66 (0.59–0.72)

CI: confidence interval.

a $\mathrm{Consistency}(\%)=\frac{\mathrm{Number of diabetes diagnosisi n both test and retest study}}{\mathrm{Number of diabetes diagnosis in test study}}\times 100$.

b Type 1 diabetes were classified as those reporting age at diagnosis <40 years.

c Type 2 diabetes were classified as those reporting age at diagnosis >39 years.

Table 4 shows the kappa agreement for the three test–retest studies separately for the two types of diabetes. The kappa agreement for type 1 diabetes was very good in the 1991–1998 test–retest study (kappa = 0.83, 95% CI: 0.76–0.89), while it was good in the 1991–2005 test–retest study (kappa = 0.76, 95% CI: 0.68–0.84), and the 1998–2005 test–retest study (kappa = 0.73, 95% CI: 0.66–0.81). The kappa agreement for type 2 diabetes was good in the 1991–1998 test–retest study (kappa = 0.67, 95% CI: 0.55–0.79), and in the 1998–2005 test–retest study (kappa = 0.66, 95% CI: 0.59–0.72), while it was moderate in the 1991–2005 test–retest study (kappa = 0.57, 95% CI: 0.43–0.71) (Table 4). The overall kappa agreement in the 1991–1998 test–retest study was stronger than in the 1991–2005 test–retest study and the 1998–2005 test–retest study (Table 4).

Table 5 presents the sensitivity of the kappa agreements by classifying those reporting age at diagnosis less than 35, as diagnosed with type 1 diabetes. While, classifying those reporting age at diagnosis greater than 44 as diagnosed with type 2 diabetes. The kappa agreements remained moderate to good for type 1 diabetes, while the kappa agreements for type 2 diabetes were fair to good (Table 5).

**Table 5.** Sensitivity analysis of kappa agreements for self-reported type 1 (age at diagnosis: <35 years) and type 2 diabetes diagnoses (age at diagnosis: >44 years).

Diabetes type	Test study	Retest study	Consistency (%)^a^	Kappa (95% CI)
	Cases in 1991 (n)	Cases in 1998 (n)		
Type 1 diabetes^b^	88	81	68/88 (77.3)	0.80 (0.65–0.95)
Type 2 diabetes^c^	12	15	6/12 (50.0)	0.52 (0.27–0.77)
	Cases in 1991 (n)	Cases in 2005 (n)		
Type 1 diabetes^b^	88	74	54/88 (61.4)	0.69 (0.51–0.88)
Type 2 diabetes^c^	12	12	3/12 (25.0)	0.33 (0.05–0.61)
	Cases in 1998 (n)	Cases in 2005 (n)		
Type 1 diabetes^b^	81	74	57/81 (70.4)	0.60 (0.38–0.81)
Type 2 diabetes^c^	129	123	75/129 (58.1)	0.63 (0.56–0.70)

CI: confidence interval.

a $\mathrm{Consistency}(\%)=\frac{\mathrm{Number of diabetes diagnosis in both test and retest study}}{\mathrm{Number of diabetes diagnosis in test study}}\times 100$.

b Only those reporting age at diagnosis <35 years were included.

c Only those reporting age at diagnosis >44 years were included.

Tables 6–10 present the kappa agreement for diabetes stratified by age, BMI, physical activity, education, and smoking status. There was no clear pattern of inconsistency in the kappa agreements within different strata of age, BMI, physical activity, and smoking (Table 6–8 and 10). However, the stratified analysis by the level of education shows that the kappa agreement is strongest among the most educated group (Table 9) in all the test–retest comparisons, while generally it was weaker among the least educated group.

**Table 6.** Kappa agreements for self-reported diabetes diagnoses (excluding gestational diabetes) stratified by age groups.

		Test study	Retest study	Consistency (%)^a^	Kappa (95% CI)
		Cases in 1991 (n)	Cases in 1998 (n)		
Age	40–44	38	31	27/38 (71.1)	0.78 (0.67–0.89)
	45–49	39	38	27/39 (69.2)	0.70 (0.58–0.82)
	50–54	59	65	48/59 (81.4)	0.77 (0.69–0.86)
	55–59	12	17	11/12 (91.7)	0.76 (0.58–0.93)
		Cases in 1991 (n)	Cases in 2005 (n)		
Age	40–44	38	30	26/38 (68.4)	0.76 (0.65–0.88)
	45–49	39	35	24/39 (61.5)	0.65 (0.52–0.77)
	50–54	59	54	34/59 (57.6)	0.60 (0.49–0.71)
	55–59	12	11	6/12 (50.0)	0.52 (0.27–0.77)
		Cases in 1998 (n)	Cases in 2005 (n)		
Age	40–44	64	57	42/64 (65.6)	0.69 (0.60–0.79)
	45–49	75	66	49/75 (65.3)	0.69 (0.61–0.78)
	50–54	143	131	98/143 (68.5)	0.71 (0.65–0.77)
	55–59	33	28	20/33 (60.6)	0.65 (0.51–0.79)

CI: confidence interval.

a $\mathrm{Consistency}(\%)=\frac{\mathrm{Number of diabetes diagnosis in both test and retest study}}{\mathrm{Number of diabetes diagnosis in test study}}\times 100$.

**Table 7.** Kappa agreements for self-reported diabetes diagnoses (excluding gestational diabetes) stratified by BMI.

		Test study	Retest study	Consistency (%)^a^	Kappa (95% CI)
		Cases in 1991 (n)	Cases in 1998 (n)		
	Normal weight (<25 kg/m^2^)	62^b^	62	49/62 (79.0)	0.79 (0.71–0.87)
BMI	Overweight (25–29.9 kg/m^2^)	44^b^	48	35/44 (79.5)	0.76 (0.66–0.86)
	Obese (⩾30 kg/m^2^)	41^b^	41	29/41 (70.7)	0.70 (0.59–0.82)
		Cases in 1991 (n)	Cases in 2005 (n)		
	Normal weight (<25 kg/m^2^)	62^b^	59^c^	44/62 (80.0)	0.73 (0.64–0.82)
BMI	Overweight (25–29.9 kg/m^2^)	44^b^	35^c^	21/44 (47.7)	0.53 (0.40–0.66)
	Obese (⩾30 kg/m^2^)	41^b^	35^c^	25/41 (61.0)	0.65 (0.53–0.78)
		Cases in 1998 (n)	Cases in 2005 (n)		
	Normal weight (<25 kg/m^2^)	99^d^	89^e^	74/99 (74.7)	0.79 (0.72–0.85)
BMI	Overweight (25–29.9 kg/m^2^)	99^d^	83^e^	59/99 (59.6)	0.65 (0.56–0.73)
	Obese (⩾30 kg/m^2^)	114^d^	106^e^	74/114 (64.9)	0.79 (0.72–0.85)

CI: confidence interval; BMI: body mass index.

a $\mathrm{Consistency}(\%)=\frac{\mathrm{Number of diabetes diagnosis in both test and retest study}}{\mathrm{Number of diabetes diagnosis in test study}}\times 100$.

b The numbers do not add up to 148 due to missing values on height or weight (consequently on BMI).

c The numbers do not add up to 130 due to missing values on height or weight (consequently on BMI).

d The numbers do not add up to 315 due to missing values on height or weight (consequently on BMI).

e The numbers do not add up to 282 due to missing values on height or weight (consequently on BMI).

**Table 8.** Kappa agreements for self-reported diabetes diagnoses (excluding gestational diabetes) stratified by physical activity.

		Test study	Retest study	Consistency (%)^a^	Kappa (95% CI)
		Cases in 1991 (n)	Cases in 1998 (n)		
	Low	24^b^	31^c^	19/24 (79.2)	0.68 (0.54–0.82)
Physical activity level	Medium	106^b^	101^c^	80/106 (75.5)	0.77 (0.71–0.84)
	High	11^b^	11^c^	8/11 (72.7)	0.73 (0.52–0.94)
		Cases in 1991 (n)	Cases in 2005 (n)		
	Low	24^b^	27^d^	18/24 (75.0)	0.74 (0.56–0.85)
Physical activity level	Medium	106^b^	86^d^	63/106 (59.4)	0.66 (0.58–0.73)
	High	11^b^	9^d^	5/11 (45.5)	0.50 (0.23–0.77)
		Cases in 1998 (n)	Cases in 2005 (n)		
	Low	62^e^	57^f^	43/62 (69.4)	0.72 (0.63–0.81)
Physical activity level	Medium	209^e^	188^f^	139/209 (66.5)	0.70 (0.65–0.75)
	High	26^e^	25^f^	17/26 (65.4)	0.67 (0.52–0.82)

CI: confidence interval.

a $\mathrm{Consistency}(\%)=\frac{\mathrm{Number of diabetes diagnosis in both test and retest study}}{\mathrm{Number of diabetes diagnosis in test study}}\times 100$.

b The numbers do not add up to 148 due to missing values on physical activity level.

c The numbers do not add up to 151 due to missing values on physical activity level.

d The numbers do not add up to 130 due to missing values on physical activity level.

e The numbers do not add up to 315 due to missing values on physical activity level.

f The numbers do not add up to 282 due to missing values on physical activity level.

**Table 9.** Kappa agreements for self-reported diabetes diagnoses (excluding gestational diabetes) stratified by education level.

		Test study	Retest study	Consistency (%)^a^	Kappa (95% CI)
		Cases in 1991 (n)	Cases in 1998 (n)		
	Primary/intermediate (0–9)	35^b^	40^c^	28/35 (80)	0.75 (0.64–0.86)
Education level (duration in years)	Secondary (10–12)	63^b^	70^c^	50/63 (79.4)	0.75 (0.67–0.83)
	University (13–16)	33^b^	24^c^	21/33 (63.6)	0.74 (0.61–0.87)
	Postgraduate and above (17+)	14^b^	15^c^	13/14 (92.9)	0.90 (0.78–1.00)
		Cases in 1991 (n)	Cases in 2005 (n)		
	Primary/intermediate (0–9)	35^b^	32^d^	18/35 (51.4)	0.54 (0.39–0.68)
Education level (duration in years)	Secondary (10–12)	63^b^	53^d^	37/63 (58.7)	0.64 (0.53–0.74)
	University (13–16)	33^b^	30^d^	24/33 (72.7)	0.76 (0.64–0.88)
	Postgraduate and above (17+)	14^b^	12^d^	10/14 (71.4)	0.77 (0.59–0.95)
		Cases in 1998 (n)	Cases in 2005 (n)		
	Primary/intermediate (0–9)	85^e^	78^f^	55/85 (64.7)	0.67 (0.59–0.75)
Education level (duration in years)	Secondary (10–12)	133^e^	112^f^	85/133 (64.0)	0.69 (0.62–0.76)
	University (13–16)	63^e^	61^f^	45/63 (71.4)	0.72 (0.64–0.81)
	Postgraduate and above (17+)	30^e^	27^f^	21/30 (70.0)	0.74 (0.61–0.86)

CI: confidence interval.

a $\mathrm{Consistency}(\%)=\frac{\mathrm{Number of diabetes diagnosis in both test and retest study}}{\mathrm{Number of diabetes diagnosis in test study}}\times 100$.

b The numbers do not add up to 148 due to missing values on education level.

c The numbers do not add up to 151 due to missing values on education level.

d The numbers do not add up to 130 due to missing values on education level.

e The numbers do not add up to 315 due to missing values on education level.

f The numbers do not add up to 282 due to missing values on education level.

**Table 10.** Kappa agreements for self-reported diabetes diagnoses (excluding gestational diabetes) stratified by smoking status.

		Test study	Retest study	Consistency (%)^a^	Kappa (95% CI)
		Cases in 1991 (n)	Cases in 1998 (n)		
	Never smoker	51	47	38/51 (74.5)	0.78 (0.68–0.87)
Smoking status	Former smoker	51	50	37/51 (72.5)	0.73 (0.63–0.83)
	Current smoker	46	54	38/46 (82.6)	0.76 (0.67–0.85)
		Cases in 1991 (n)	Cases in 2005 (n)		
	Never smoker	51	41	29/51 (56.9)	0.63 (0.51–0.75)
Smoking status	Former smoker	51	40	31/51 (60.8)	0.68 (0.57–0.79)
	Current smoker	46	49	30/46 (65.2)	0.63 (0.52–0.74)
		Cases in 1998 (n)	Cases in 2005 (n)		
	Never smoker	108	94	72/108 (66.7)	0.71 (0.64–0.78)
Smoking status	Former smoker	103	93	62/103 (60.2)	0.63 (0.55–0.71)
	Current smoker	104	95	75/104 (72.1)	0.75 (0.68–0.82)

CI: confidence interval.

a $\mathrm{Consistency}(\%)=\frac{\mathrm{Number of diabetes diagnosis in both test and retest study}}{\mathrm{Number of diabetes diagnosis in test study}}\times 100$.

## Discussion

In this study, we analyzed the test–retest reliability of self-reported diabetes diagnosis in a large sample of middle-aged women in Norway. We observed that the agreement was good for all diabetes diagnoses combined in all three test–retest studies. The weakest agreement was found in the 1991–2005 test–retest study. This was to be expected, as the time interval between these studies was the longest. These results also suggest that other confounding factors may have affected self-reported diabetes diagnosis in the 1991–1998, or 1998–2005 test–retest studies, as the agreement in these periods was expected to be more similar. The fact that diabetes diagnosis may change over time could have contributed to the decreasing agreement observed between the 1991–1998 test–retest study and the 1991–2005 test–retest study. However, looking at the two types of diabetes separately revealed some differences in the kappa agreement. The kappa agreement for type 1 diabetes was weakest in the 1998–2005 test–retest study, which was very close to the kappa agreement for the ~14-year interval in the 1991–2005 test–retest study. In summary, the results show that although the agreement for all self-reported diabetes was weakest in the 1991–2005 test–retest study, this was not the case when analyzing the kappa agreement for the two types of diabetes separately. This suggests that recall problems may not be an important determinant of the accuracy of self-reported diabetes diagnosis.

One possible reason for the higher kappa agreement among women with type 1 diabetes in our study is that these women may have severe complications sooner^64^ than women with type 2 diabetes; this may have contributed the women’s recall of age at diagnosis, resulting in a higher agreement for type 1 diabetes.

Since type 2 diabetes typically affects people 40 years of age and over,^51,52^ we classified only women aged 40 years and over as having type 2 diabetes. However, it is still possible that women younger than 40 years of age have developed type 2 diabetes.^65–69^ In addition, cases identified as having gestational diabetes were excluded from the type 2 diabetes group, although women who had gestational diabetes may develop type 2 diabetes later in life.^70,71^ Women aged 39 years or less who reported a diabetes diagnosis (excluding gestational diabetes) were categorized as having type 1 diabetes. Since type 1 diabetes can occur at any age,^72^ it is also possible that some of the women classified as having type 2 diabetes in fact had type 1 diabetes. Due to the design and self-reported nature of the study, it was not possible to confirm the exact type(s) of diabetes diagnosis. The results from sensitivity analysis restricting type 1 diabetes cases to those reporting age at diagnosis less than 35 years, and restricting type 2 diabetes to those reporting age at diagnosis more than 44 years, were still acceptable.

This study was larger than previous studies, permitting subgroup analyses. No clear pattern of inconsistency in kappa agreements was observed between different strata of BMI, physical activity, and smoking status. Although no formal test of heterogeneity was performed to assess the statistical difference in kappa agreements across the subgroups, there was a pattern across education groups. The kappa agreement was strongest among the most educated group, while generally it was weaker among the least educated group.

Although the NOWAC cohort is representative of Norwegian women in corresponding age groups, the current sample may not be a representative sample since it includes only the women participating in all the three waves of the study. Furthermore, the respondents with missing values were excluded. Some research suggests that those belonging to the low socio-economic strata, and are relatively unhealthy, are likely to have a higher proportion of missing values in observational study.^73^ Multiple imputation (MI) was not performed, since the kappa statistic^61^ is not supported with MI software’s^74–77^ in Stata. Therefore, the possibility of selection bias limits the external validity of this study.

The kappa agreement we report here is not comparable to other studies^63,78^ due to differences in the proportion of people reporting a certain type of diabetes in different studies, or differences in distribution. We found few studies assessing the test–retest reliability of diabetes diagnosis, and the results of those that were found were not consistent. Most showed very good agreement^45–49,79^ between the test and the retest studies, while others showed a good^50^ or moderate^80^ level of agreement. However, most of the studies we found^46–49,80^ did not report either the significance probability or the CIs. One possible reason for the higher kappa agreement reported in previous studies^45–50^ may be the relatively small time interval between the test and retest studies, as compared to the ~7- or ~14-year interval in our study. The relatively smaller time interval between the test and retest studies may have caused respondents in other populations to remember their previous response more easily, resulting in a higher kappa coefficient.

Another key difference between previous studies^45–50^ and our study was their use of interview to collect the information on diabetes diagnosis. As these studies used an interview setting, it is reasonable to assume that the respondent had a chance to ask for questions to be repeated, or for further explanation/clarification, and that the interviewer might have provided it. This may have helped the respondents to understand the question better, and to therefore report more accurately. It is probable that this key difference in the investigation tool increases the kappa agreement for the test–retest reliability of the studies using interviews to collect data.

However, a study from Manhattan (New York)^80^ reported on the test–retest reliability of diabetes diagnosis using telephone interviews. The retest study was conducted within 30 days of the test study, and the kappa agreement between the test and retest studies was found to be 0.48, which is very low considering the short time interval, and despite the use of interviews to collect data. This shows that a short time interval between the test and the retest study and the use of interviews do not necessarily increase the kappa agreement.

The strength of this study is that, it is the first to assess the test–retest reliability of self-reported diabetes diagnosis separately for type 1 and type 2 diabetes. Other strengths of our study include a large cohort size, sensitivity of the estimates by self-reported age at diagnosis, and subgroup analysis within different covariates. This study provides new insights into earlier research by providing the reliability of self-reported diagnosis separately for type 1 and type 2 diabetes.

## Strengths and limitation of this study

- Large (n = 33,919) longitudinal population-based study.
- First to assess the test–retest reliability of self-reported diabetes diagnosis separately for type 1 and type 2 diabetes.
- Some women younger than 40 years of age may have developed type 2 diabetes.
- Women with gestational diabetes were excluded, although they may develop type 2 diabetes later in life.

## Conclusion

In conclusion, this study shows that the reliability of the self-reported information on diabetes diagnosis from a large prospective cohort study with long time interval is satisfactory.
